# An Intelligent Model-Based Effective Approach for Glycemic Control in Type-1 Diabetes

**DOI:** 10.3390/s22207773

**Published:** 2022-10-13

**Authors:** Ali Khaqan, Ali Nauman, Sana Shuja, Tahir Khurshaid, Ki-Chai Kim

**Affiliations:** 1 Department of Electrical Engineering, COMSATS University Islamabad, Islamabad 45550, Pakistan; 2 Department of Information and Communication Engineering, Yeungnam University, Gyeongsan 38541, Korea; 3 Department of Electrical Engineering, Yeungnam University, Gyeongsan 38541, Korea

**Keywords:** type-1 diabetes mellitus (T1DM), glycemic targets, closed-loop insulin infusion systems, continuous glucose monitoring (CGM)

## Abstract

Type-1 diabetes mellitus (T1DM) is a challenging disorder which essentially involves regulation of the glucose levels to avoid hyperglycemia as well as hypoglycemia. For this purpose, this research paper proposes and develops control algorithms using an intelligent predictive control model, which is based on a UVA/Padova metabolic simulator. The primary objective of the designed control laws is to provide an automatic blood glucose control in insulin-dependent patients so as to improve their life quality and to reduce the need of an extremely demanding self-management plan. Various linear and nonlinear control algorithms have been explored and implemented on the estimated model. Linear techniques include the Proportional Integral Derivative (PID) and Linear Quadratic Regulator (LQR), and nonlinear control strategy includes the Sliding Mode Control (SMC), which are implemented in this research work for continuous monitoring of glucose levels. Performance comparison based on simulation results demonstrated that SMC proved to be most efficient in terms of regulating glucose profile to a reference level of 70 mg/dL compared to the classical linear techniques. A brief comparison is presented between the linear techniques (PID and LQR), and nonlinear technique (SMC) for analysis purposes proving the efficacy of the design.

## 1. Introduction

Patients having type 1 diabetes suffer from decrements in the required insulin level as a result of eradication of beta cells. Type 1 Diabetes Mellitus (T1DM) clinically appears before the age of 35 years [1]. This type of diabetes leads to hyperglycemia, which can cause further problems due to high blood glucose sugar [2]. Consequently, insulin has to be supplied in order to avoid complications, including dehydration and death [3]. Keeping an adequate level of insulin in the body is pivotal to cope with T1DM. Automatic blood glucose control in diabetic patients can improve their life and also reduce the expenditure on their treatment.

Both intelligent model-based and model-independent strategies are possible for designing an optimal controller to reduce the risk of diabetes. The latter empirical strategy does not rely on an analytical model of the glucose–insulin setup, thereby providing a control law to regulate insulin infusion using pre-available data from experiments [4]. In this regard, application and improvements of Proportional Integral Derivative (PID) controllers for regulating insulin infusion rate have been still in vogue [5]. In contrast, the strategy based on a model exploits a priori knowledge of physiological parameters. Control problem can therefore be analytically formulated in an optimal way. Technological advancements related to infusion sets and insulin pumps have improved with the progression in a variety of different designs when compared with conventional insulin injections. In [6], the authors reviewed and compared the preceding analysis and performance with the existing CSII pumps in the type 1 diabetic patients. An artificial pancreas consists of a pump for Continuous Subcutaneous Insulin Infusion (CSII), a setup for uninterrupted glucose monitoring and a control law to realize a closed-loop feedback system. In [7], authors discussed the non-trial treatment for type 1 diabetic patients using Do-It-Yourself Artificial Pancreas systems (DIY-APS), which is an open source and hybrid closed-loop system. The research work amalgamates the promising and upcoming clinical literature related to DIY-APS with the point of views suggested by different healthcare professionals and DIY-APS users to improve the daily routine plan for type 1 patients in a real-life scenario. Authors presented a detailed discussion regarding the advancements in the closed-loop design of systems for efficient handling of patients with diabetes. The study focused on the working of artificial pancreas demonstrating the improvement in glycemic effects and lessening the load of a hectic self-management plan in daily life for diabetic patients [8]. 

The classical model for blood glucose control describes glucose–insulin kinetics with the help of nonlinear differential equations. Proposed in the early 1980s, it was a single compartment model based on conceptual compartmentalization of insulin storage. In [9], the authors presented and discussed the pros and cons of the different insulin delivery systems designed via closed-loop configuration for examining the blood glucose levels. The research study covered the concepts from early technologies including syringes, needles to bionic pancreas and (DIYAPS). For the injection of insulin, it is important to take note of exercise routine, current glucose levels and the idea of food intake as it affects glucose levels considerably. Authors suggested a design of mobile application for diabetic patients based on mathematical models illustrating the effects of blood–glucose concentration. The proposed application caters the effects produced during the process of meal intakes and exercise periods on the blood–glucose concentration [10]. A two-compartmental model is designed using a minimal model for plasma–glucose concentrations for healthy and T1D patients [11]. In a study, authors presented an alternative approach for subcutaneous insulin infusion. Regardless of the current technological advancements, the aforesaid scheme has a limited scope for insulin control. This research study deals with the design of Intraperitoneal (IP) insulin control for the distribution and absorption of insulin [12]. 

Various control algorithms based on a variety of these mathematical models have been reported for optimization of insulin therapy procedures in patients suffering from diabetes. Some of these developments are based on the Proportional Integral Derivative (PID), which is widely used in control applications due to its inherent capability to obtain quick transient response in terms of achieving a steady state in less time and to reduce steady-state error. In [13], a PID controller is used for the infusion of insulin and glucagon to control the glucose levels in the patient’s body using artificial intelligence and predictive control resulting in the lower range of glucose concentration. In [14], two different control strategies, MPC and PID, are evaluated to control the glucose levels in patients affected from exercise and meal intakes to avoid hypoglycemia. Pole placement and optimal control algorithms have also been applied for insulin infusion. Moreover, a number of Model Predictive Control (MPC) laws have been realized and tested in clinical trials which show better results in managing constraints on the controlled and manipulated variables in a systematic manner. A novel strategy was designed in [15] to regulate the parameters of insulin-on-board (IOB) in a real-world scenario. The suggested design was distinctly completed using two different approaches for fasting periods and postprandial periods. The results obtained using the designed technique showed glucose levels in the desired range maintaining the patient’s safety and health. Furthermore, control techniques like Linear Parameter Varying (LPV)-based laws offer a multivariable controller to ensure robustness and to address bandwidth limitations. In [16], a linear filter is proposed using a LPV-based controller to achieve the required plasma–glucose concentrations showing the efficacy of the design for insulin infusion. Another control technique, H-infinity, offers high disturbance rejection and more stability due to its nonlinear nature. [17], showed that large nonlinearity and uncertainty involved in the mathematical representation of the insulin metabolism process lead to the design of the H-infinity controller. The controller was designed to regulate the plasma–glucose concentration for T1DM patients by minimizing the magnitude of the H-infinity norm with respect to external disturbances present in the closed-loop system.

Fuzzy-logic theory has also been applied for insulin infusion. A Mamdani-type fuzzy control scheme is used in a closed-loop manner to maintain the glucose levels in type 1 diabetic patients. Simulation results showed that the designed control algorithm can be used successfully for designing the artificial pancreas [18].

Due to the linear nature of most of the control strategies discussed above, these techniques are incapable of adequately dealing with uncertainties and disturbances which are inherently present in the real-world systems. The present study aimed to design control strategies based on the Linear Quadratic Regulator (LQR) and Sliding Mode Control (SMC), which according to our knowledge, has not been applied comprehensively and comparatively on the estimated model reported in [19]. PID has been chosen as a classical benchmark for performance comparison. LQR is a linear control method which is well known for its feature of decreasing the control effort to optimize the designed controller by an appropriate choice of Q and R matrices [20]. The reliability of the control law designed in LQR is a function of these matrices and may require different values of the matrices in different control scenarios. For the systems with unmodeled disturbances and parametric variations, the SMC technique is an attractive control method due to its robust nature and disturbance rejection capability [21]. SMC was selected due to its robustness, finite-time convergence, ultimate accuracy and insensitive nature to external and internal disturbances [22]. Consequently, SMC serves as an appropriate choice for physiological settings requiring high precision. The emphasis of this research article is on the control design to regulate the insulin infusion using linear and nonlinear control techniques. The controller designed through the nonlinear control technique, SMC, achieves the desired glucose levels (70 mg/dL) in minimum time, proving the efficacy of the design. Simulation results obtained with the SMC controller show better performance in terms of their time domain specifications when compared with linear (PID and LQR) and nonlinear transformation-based SMC controllers available in literature.

The paper is organized in six sections. The dynamic model of a glucose insulin system is derived in Section 2. The theoretical basis of a controller design and functions are discussed in detail in Section 3. Section 4 presents the simulation results and discussion. Section 5 shows the comparison between two linear control techniques (PID and LQR) used in this research work and a nonlinear control technique Sliding Mode Control (SMC). Finally, Section 6 concludes the paper.

## 2. Modeling and Analysis

### 2.1. The Estimated Model

The estimated model approach to describe system dynamics of glucose insulin relies on data extracted from sources such as research studies, hospitals and diabetes simulators, e.g., Attention Interest Desire Action (AIDA), UVA/Padova, ESIM simulator, etc. In the present research, an estimated model has been derived using a UVA/Padova simulator. The identification procedure is detailed below: for each in silico patient, the deviation of insulin delivery from the desired glucose concentration is identified by a linear model. To ensure capturing of the response at various points, interstitial concentrations of 90, 120 and 150 mg/dL of glucose have been taken. Thus, three linear models corresponding to each patient have been obtained. For a particular concentration of glucose, the identification process consists of obtaining the basal insulin (Ib) at steady state and then adding it to a sinusoidal insulin sweep. The glucose deviation is finally captured by infusing the signal obtained in the previous step. Subspace identification algorithms have been used to obtain third-order models in all the cases as in [23].

**Figure 1:**
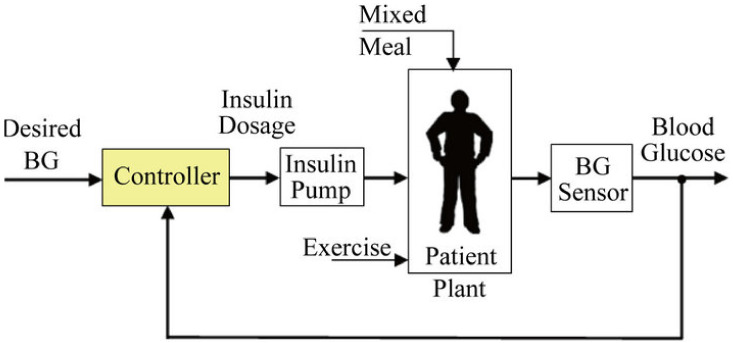
Conceptual diagram of closed-loop control scheme.

For control purposes, a third-order discrete transfer function $G\left(z\right)$ relating glucose to insulin has been defined as,

(1) $$G\left(z\right)=-\frac{C_{0}z^{-3}}{\left(1-z^{-1}p_{1}\right)\left(1-z^{-1}p_{2}\right)\left(1-z^{-1}p_{3}\right)}$$

where $p_{1}$,$\;p_{2}$, and $p_{3}$ vary from patient to patient [19]. Figure 1 shows the basic diagram of the feedback control system.

This model given in (2) has been transformed into a continuous model using zero-order hold for computational convenience, as shown in (3). The following transfer function has been obtained by using values of parameters $C_{o\;}$, $p_{1}$, $p_{2}$ and $p_{3}$ as 0.132, 0.965, 0.95 and 0.93, respectively. The values are chosen such that the gain is overestimated and the phase is kept lower than all other identified models in order to obtain robustness.

(2) $$G\left(z\right)=-\frac{0.132}{\left(z-p_{1}\right)\left(z-p_{2}\right)\left(z-p_{3}\right)}$$

(3) $$G\left(s\right)=\frac{-8.263\left(10^{-5}\right)s^{2}+4.022\left(10^{-7}\right)s-6.61\left(10^{-10}\right)}{s^{3}+2.658\left(10^{-4}\right)s^{2}+2.26\left(10^{-8}\right)s+6.14\left(10^{-13}\right)}\;$$

Using (3), the system model in state space representation has been derived as,

(3a) $$A=\left[\begin{array}{ccc} 2.805 & -1.311 & 0.8172\\ 2 & 0 & 0\\ 0 & 0.5 & 0 \end{array}\right]\;\;\;\;\;\;\;\;\;\;$$

(3b) $$B=\left[\begin{array}{c} 0.5\\ 0\\ 0 \end{array}\right]\;\;$$

(3c) $$C=\left[\begin{array}{ccc} 0\;\; & 0\;\; & -0.264 \end{array}\right]$$

(3d) $$D=\left[0\right]\;$$

where A is the state matrix, B is the input matrix, C is the output matrix and D is the direct transmission matrix as shown in (3a) to (3d).

### 2.2. Closed-Loop Response of the Estimated Model

**Figure 2:**
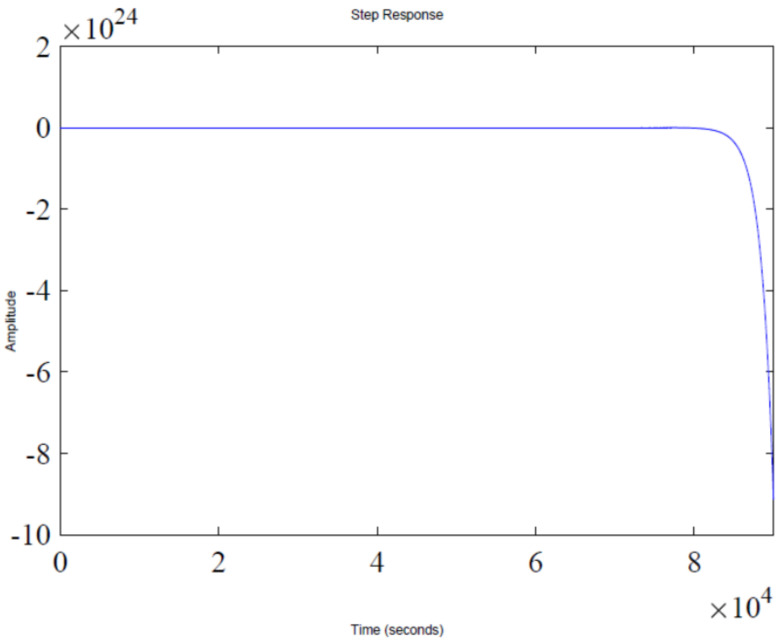
Closed-loop unit step response.

**Figure 3:**
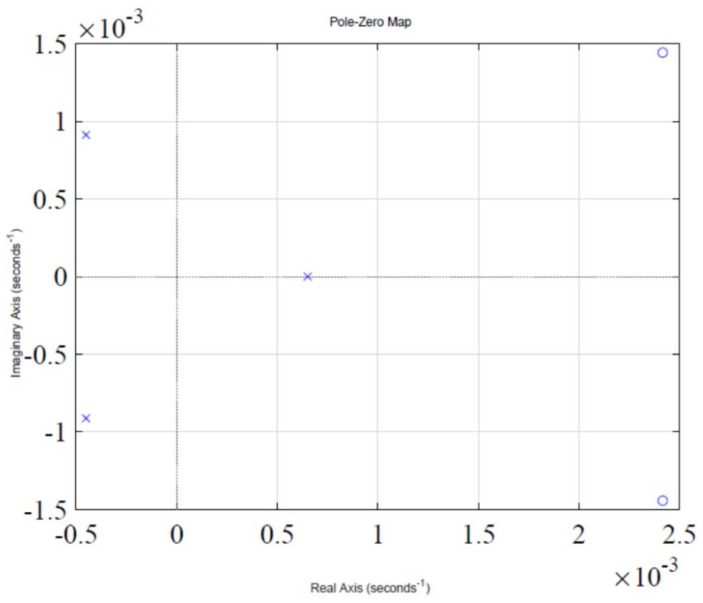
Pole-zero plot of the system.

The step response of the feedback loop system is shown in Figure 2. It is evident that system response leads to instability, which is due to presence of a pole in the Right Half-Plane (RHP), as shown in Figure 3, which illustrates a pole-zero plot. Thus, a control algorithm has to be designed that could shift the RHP pole to the Left Half-Plane (LHP), subsequently making the system response stable.

### 2.3. Controllability and Observability

The matrices ⏀ and ᴪ, (3e) and (3f), respectively indicate controllable and observable notion of the system that have been calculated by using system matrices A, B and C in ⏀ = [$B,AB,A^{2}B,A^{3}B,A^{n-1}B$] and ᴪ =${\left[C,CA,CA^{2},CA^{3}\ldots .CA^{n-1}\right]}^{T}$, where “n” is the rank of matrix A. The resultant matrices given below are full-rank matrices; therefore, all states are completely controllable and observable, which implies that a state feedback controller can be designed.

(3e) $$⏀=\left[\begin{array}{ccc} 0.5 & 1.4025 & 2.6228\\ 0 & 1 & 2.805\\ 0 & 0 & 0.5 \end{array}\right]$$

(3f) $$ᴪ=\left[\begin{array}{ccc} 0 & 0 & -2.64\\ 2 & 0.132 & 0\\ -2.64 & 0 & 0 \end{array}\right]\;\;$$

In the following section, we have shown the details of a linear (PID and LQR) and nonlinear control technique (SMC), which we will be using to design the control laws to maintain the blood glucose level within the range.

## 3. Controller Design Methods

Over- and under-dosing of insulin in diabetics have some serious effects on their health. The major task in glycemic control is to infuse the right quantity of insulin to ensure that the glucose level remains within the standard physiological range (70 to 110 mg/dL). Both linear and nonlinear control design methods have been employed on estimated models to control the glucose level given in Section 2. In the next section, a detailed and comprehensive discussion is presented for the linear and nonlinear control methods studied in this research work.

### 3.1. Linear Control Algorithms

Systems obeying a superposition principle are mainly controlled by linear control algorithms, as any change in system input would cause an equal amount of change in the output [24]. While using linear control algorithms, model uncertainties and disturbances like exercise and meal are taken as zero. In this research work, two linear control techniques, PID and LQR, are implemented for glycemic control. The objective is to adjust the amount of insulin delivery so that it provides good control of glucose levels in less amount of time.

#### 3.1.1. PID Control

PID controllers are widely used in industrial applications as well as in many research areas because of their efficient nature, simplicity, fast response and having large power to overcome the steady-state error. PID controllers are the typical option to start with, and reasonable results may be obtained. The quick response or the speed of the response is the main advantage of PID control. The PID controllers are involved in three types of control actions, proportional control, integral control, and derivative control, where $k_{p\;},\;k_{i}\;\mathrm{and}\;k_{d\;}$are the individual gains of these control actions, respectively [25]. In PID, the system output is connected with the input. If the output of the system has some difference between the reference point, then PID controllers modify the input according to the difference in the reference point and the current output. The output of the system due to the proportional parameter of the controller depends on the present change in the error, output due to the integral parameter depends on the past error and the output due to the derivative parameter depends on the future error.

In order to get the desired output of the system in terms of transient and steady-state response, we usually adjust or tune the values of these three parameters. The PID controller operates on the error signal *e*(*t*) to regulate the overall control action of the system. In this control law, the control input which minimizes the error is the weighted sum of three terms, i.e.,

(4) $$u\left(t\right)=k_{p}e\left(t\right)+k_{i}\int e\left(t\right)dt+k_{d}\frac{de\left(t\right)}{dt}\;$$

and its s-domain transfer function is shown in (5) as,

(5) $$H_{c}\left(s\right)=k_{p}+\frac{k_{i}}{s}+sk_{d}\;\;\;\;\;\;\;\;$$

$k_{p}$ represents the present gain of the error, $k_{i}$ represents the past error and $k_{d}$ represents the future error, 't' is instantaneous time, $e\left(t\right)$ is the difference between measured and desired glucose values and $u\left(t\right)$ is the control input. Table 1 shows the effect of different gains of the PID controller.

#### 3.1.2. Linear Quadratic Regulator

This is an optimal control law which serves as a basis for robust techniques. Systems with conventional LQR controllers present good stability properties and are optimal with respect to a certain performance index. However, LQR control does not assure robust stability when the system is highly uncertain. The classic LQR approach deals with the optimization of a cost function or performance index. Thus, the designer can weight which states and which inputs are more important in the control action to seek for appropriate transient and steady-state performances. The main objective in this method is to minimize quadratic cost function given using,

(6) $$J\left(x,t\right)=\int \left(x^{T}Qx\left(t\right)f\left(t\right)+u^{T}Ru\right)dt\;$$

where$\;R=R^{t}>0$, $Q=Q^{t}\geq 0$ are matrices satisfying positive definite property. The LQR problem can be viewed as the weighted minimization of a linear combination of the states “*x*” and the control input u. The cost function, defined by quadratic equation, is minimum where the dynamic system is described by a linear differential equation. The Q matrix deals with the bad performance of the system and the R matrix deals with how much the effort is required in the system. The integral part shows how quickly the system returned to its normal position. In order to calculate the gain matrix K, the following relation has been used,

(7) $$K=R^{-1}B^{T}P\;$$

where 'P' also satisfies the positive definite property. 

Proper functioning of the control systems designed through linear control approaches heavily rely on the fact that the model parameters to be analyzed should be valid in the small operating range. Because of such limitations, systems designed with linear strategies might perform inadequately or often become unstable, staying far from the required specifications. Linear controllers are not sufficiently skilled to handle uncertainties and nonlinearities influencing the system. Contrary to linear control strategies, nonlinear control methods are very useful under circumstances where uncertainties and nonlinearities have a pivotal role in the system. Nonlinear controller design techniques accommodate the uncertainties and nonlinearities existing in the system and achieving a stable state efficiently. In the next subsection, a brief theoretical background related to the Sliding Mode Control (SMC) is presented for controller design.

#### 3.1.3. Nonlinear Control Method

Nonlinear control methods are applicable on the systems which are time-variant, nonlinear in behavior, or both. This theory is generally applied to real-world control systems owing to their inherently nonlinear nature [26,27]. The major advantage of this theory is that it ensures robustness against uncertainties and disturbances. Nonlinear control strategies may lead towards the simpler controller design when compared to its corresponding linear part while having the superior performance. In practical scenarios with linear techniques, high-quality sensors and actuators are needed to generate and record the linear behavior for the proper functioning of the control system in the desired range, whereas a similar control system designed through nonlinear method may permit the designer to use the cheaper components having nonlinear properties.

In this research work, a nonlinear control strategy, the Sliding Mode Control (SMC), is used to design the control law to regulate the glucose level in a diabetic patient. The robust nature of the Sliding Mode Control to cater to the effects of external disturbances acting on the system inspired the control engineers to explore this technique for diverse applications, especially in the biomedical field.

SMC is a nonlinear control method which changes the nonlinear dynamics and ensures robustness [28,29]. The basic principle of SMC is to changes system dynamics in such a way that states remain on a sliding manifold, as parameters are varied by the controller. The main property of this design technique is the enforcement of the states of the system trajectories onto a defined surface, resulting in a reaching phase [30]. These surfaces are also known as manifold and created as some hypersurface or intersection of hypersurfaces in the state space known as switching surfaces. As the states of the system reach the switching surface, the structure of the feedback loop is adaptively altered to slide the system states along the switching manifold, and this phase is known as the sliding phase.

While using SMC for glycemic control, the primary objective is to ensure robustness against disturbances such as exercises and meals. In this regard, a sliding surface 's' comprising of system states is designed.

In this procedure, the system states move from the non-zero initial state towards the sliding surface and stay on that surface for all future values of time. The designed control law consists of two parts: $u_{eq}$ and $u_{disc}$, corresponding to the reaching phase and sliding phase respectively, where $u_{eq}$ and $u_{disc}$ are the equivalent control and discontinuous control, respectively. The control law designed by using the sliding mode control is given as,

(8) $$u=u_{eq}+u_{disc}$$

$u_{eq}$ is designed by putting the derivative of sliding surface equal to zero. $u_{disc}$ is obtained with such conditions that it rejects the effect of the disturbance terms and maintains the derivative of the sliding surface equal to zero. Usually,

(9) $$u_{disc}=-ksign\left(s\right)\;\;\;$$

For stability purposes, the Lyapunov candidate function is defined as,

(10) $$V=\frac{1}{2}(s^{2})\;\;\;\;\;\;$$

with 's' being sliding surface and this Lyapunov function is positive definite if and only if,

(11) $$\dot{V}=s\dot{s}\leq 0\;$$

Section 4.3 shows in detail the steps needed to design a Sliding Mode Controller. In the following section, a detailed discussion is presented on the simulation results obtained using PID, LQR and SMC for analysis and comparison purposes.

## 4. Results and Discussion

**Figure 4:**
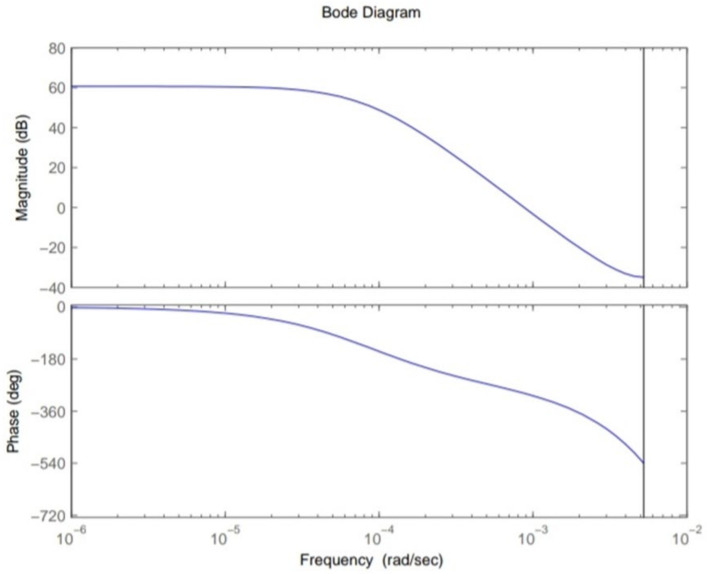
Bode plot of system.

**Figure 5:**
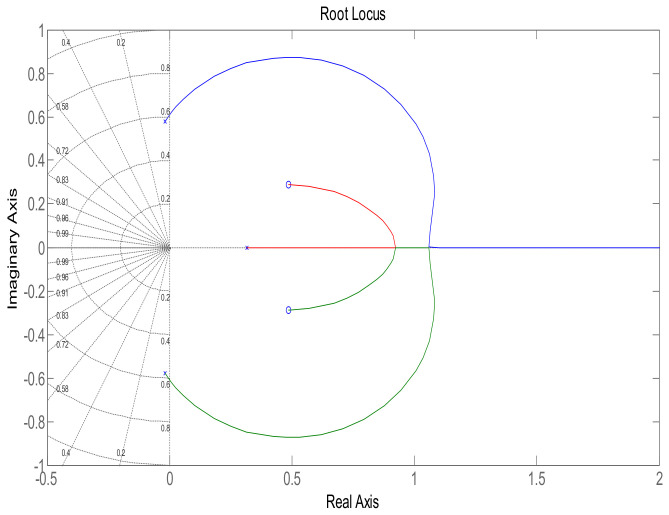
Root locus of system.

**Figure 6:**
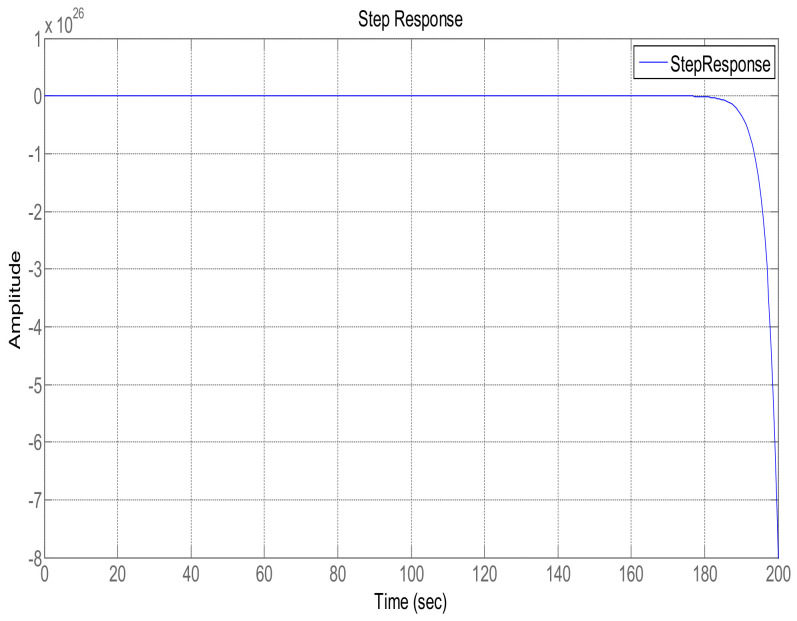
Step response of the system.

This section shows the design with simulation results of each controller studied in this research work in maintaining the glucose concentration level within normal range for diabetic patients in detail. To investigate the performance of a feedback-controlled insulin infusion system, the predefined model shown in (1) is analyzed individually. Simulation results of the system without a controller in terms of its bode plot, root locus and step response are shown in Figure 4, Figure 5 and Figure 6. The frequency response shows that the system is not stable, as well as the root locus in which the poles are in Right Half-Plane. Similarly, the step response shows that the system is not following the step input. These results reflect clearly the unstable nature of the system which demands the need of a controller that can handle and control the insulin infusion system for the adequate supply of insulin to the patients.

### 4.1. PID Controller

**Figure 7:**
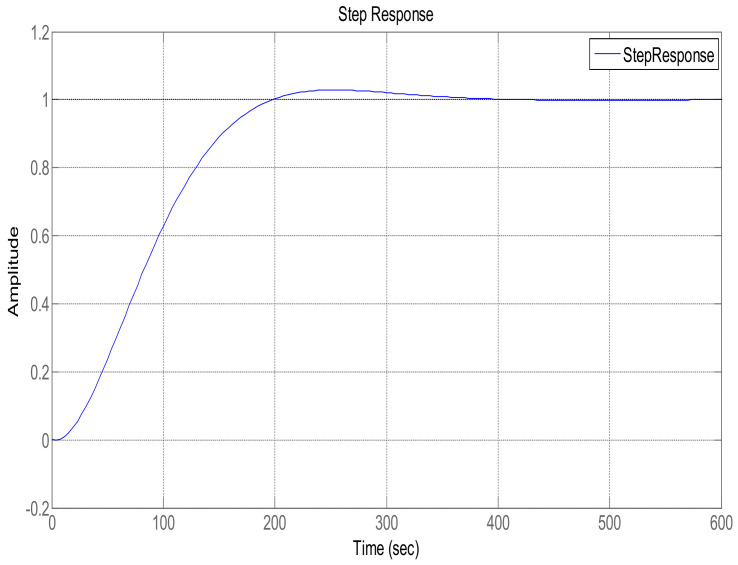
Step response using the PID controller.

**Figure 8:**
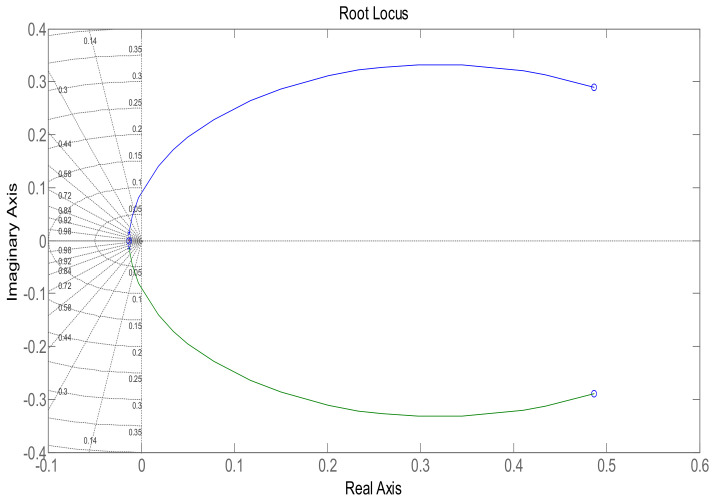
Root locus using the PID controller.

**Figure 9:**
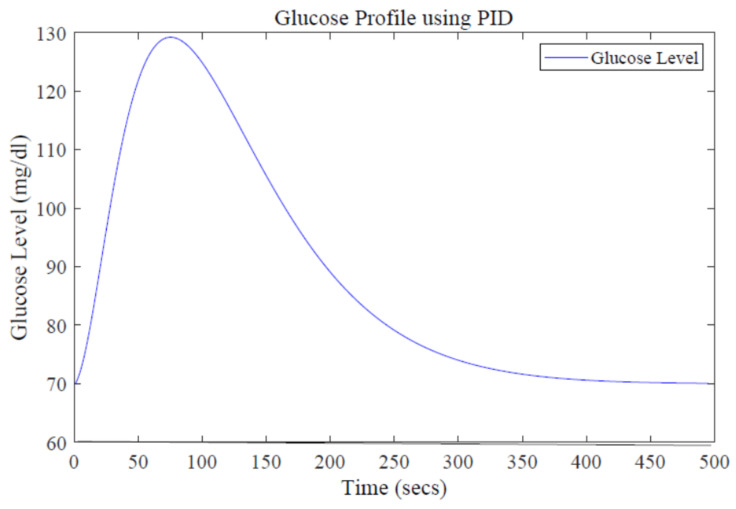
Glucose profile using the PID design.

In order to maintain glucose concentration within a normal range in the human body, a Proportional, Integral, Derivative control-based technique is designed and implemented. The PID controller is successfully implemented to control the concentration of glucose level in blood. The step response of the system follows the step input with the PID controller, showing the stability of the insulin infusion system, as depicted in Figure 7. Similarly, the root locus of the system in the presence of a designed PID controller shows that the poles move towards the Left Half-Plane, as shown in Figure 8. The tuning parameters for the PID controller are chosen as follows, and are shown in Table 2. The glucose profile shown in Figure 9 reveals that, by using the PID controller, the glucose level is maintained in the defined range and it can be interpreted that a glucose level of 70 mg/dL is obtained after approximately 430 s, thus demonstrating very large settling time.

### 4.2. Linear Quadratic Regulator

Since states are controllable and observable, choosing R=I and Q matrix as under,

(12a) $$Q=\left[\begin{array}{ccc} 0.001 & 0 & 0\\ 0 & 0.2341 & 0\\ 0 & 0 & 0.22 \end{array}\right]\;$$

The gain matrix 'k' is found to be,

(12b) $$K=\left[\begin{array}{ccc} 0.231 & 0.4947 & 0.4687 \end{array}\right]$$

**Figure 10:**
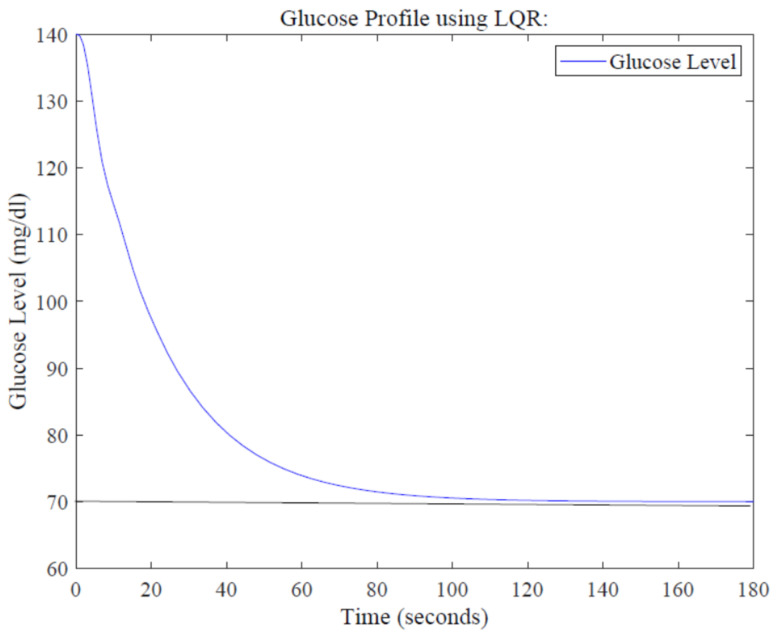
Glucose profile using the LQR design.

Simulation results for the glucose profile using LQR are shown in Figure 10. It is evident that the same glucose level of 70 mg/dL is achieved at around 113 s, showing better performance compared to the PID controller in terms of settling time.

A major drawback of a linear control algorithm is that it cannot perform adequately in the presence of disturbances and uncertainties. The uncertainties appearing in the system may be caused by variations in parameters, unmodeled dynamics and internal and external disturbances. These limitations and poor performance in linear control techniques motivated us to apply a nonlinear control method on the same model, since nonlinear techniques overperform compared to their linear counterparts, as highlighted in [31,32].

### 4.3. Sliding Mode Control

The design of a control law based on SMC requires definition of a sliding surface shown as,

(13) $$s=c_{1}x_{1}+c_{2}x_{2}+c_{3}x_{3}\;$$

where $c_{1},\;c_{2}\;\mathrm{and}\;c_{3}$ are tuning parameters. With $c_{1}$ and $c_{3}=1$, the values of other parameters are chosen in a way that the derivative of the sliding surface becomes Hurwitz monic-polynomial. This condition ensures reduction in order of the system, which can be represented with n−1 states. Such a system demonstrates insensitivity to matched uncertainties. Taking the derivative of Equation (13) wrt time 't', we get,

(14) $$\dot{s}=c_{1}\dot{x_{1}}+c_{2}\dot{x_{2}}+c_{3}\dot{x_{3}}$$

Now, let us define a positive definite Lyapunov function as under,

(15) $$V=\frac{1}{2}\left(s^{2}\right)$$

Differentiating wrt time 't', we obtain,

(16) $$\dot{V}=s\dot{s}\leq 0$$

Using the value of $\dot{s}$ in (16), we get,

(17) $$\dot{V}=s(c_{1}\dot{x_{1}}+c_{2}\dot{x_{2}}+c_{3}\dot{x_{3})}$$

From state equations, we have,

(18) $$\begin{array}{c} \;\dot{V}=\left(c_{1}x_{1}+c_{2}x_{2}+c_{3}x_{3}\right)(2.416\left(10^{-4}\right)x_{1}+5.7105\left(10^{-4}\right)x_{2}-9.105\left(10^{-5}\right)x_{3}\\ +0.25u) \end{array}$$

The equivalent control forces the system dynamics to move to the sliding surface and depends on the states of the system and state parameters. It makes the derivative of sliding manifold equal to zero. Therefore, taking $\dot{s}=0$, the control input 'u' is defined as,

(19) $$\left(2.416\left(10^{-4}\right)x_{1}+5.7105\left(10^{-4}\right)x_{2}-9.105\left(10^{-5}\right)x_{3}+0.25u\right)=0\;\;$$

(20) $$\begin{array}{c} u_{eq}=\left(\frac{1}{0.25}\right)(-2.416\left(10^{-4}\right)x_{1}-5.7105\left(10^{-4}\right)x_{2}\\ +9.105\left(10^{-5}\right)x_{3} \end{array}$$

The overall control law 'u' designed through SMC consists of equivalent control $\left(u_{eq}\right)$ and discontinuous control $\left(u_{disc}\right)$, i.e.,

(21) $$u=u_{eq}+u_{disc}$$

The designed control law including $u_{disc}$ can be written as,

(22) $$u_{eq}=\left(\frac{1}{0.25}\right)(\left(-2.416\left(10^{-4}\right)x_{1}-5.7105\left(10^{-4}\right)x_{2}+9.105\left(10^{-5}\right)x_{3}+u_{disc}\right)\;\;$$

where $u_{disc}=-k_{1}s-k_{s}Sign\left(s\right)$ is the discontinuous control, and $k_{1}$ and $k_{2}$ are control parameters for tuning, where $u_{disc}$ can be completely defined using signum function as,

(23) $$Sign\left(s\right)=\left[\begin{array}{c} +1\;\;for\;\;s>0\\ -1\;\;for\;\;s<0 \end{array}\right]$$

Thus, we can write,

(24) $$\dot{V}=s(-k_{1}\dot{s}-k_{2}sign\left(s\right)$$

(25) $$\dot{V}=-k_{1}s^{2}-k_{2}\|s\|$$

This relation shows that the Lyapunov function is negative-definite. Equation (22) shows the derived control law using the nonlinear control strategy (SMC). To investigate and characterize the performance of the designed controller, the resultant control law is implemented in MATLAB, which shows better results as compared to the linear control strategies (PID and LQR) in terms of transient and steady-state parameters. Table 3 lists values of various parameters used in the design of SMC-based law.

**Figure 11:**
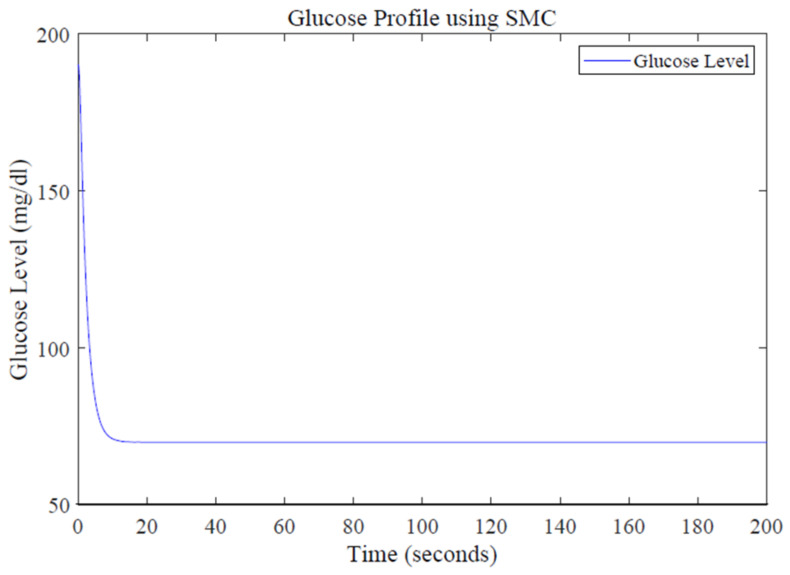
Glucose profile using the SMC design.

A reference glucose level of 70 mg/dL is defined using MATLAB function. The quantity of infused insulin is determined by the designed control law. Simulation results for glucose profile using SMC are shown in Figure 11.

The designed controller efficiently tracks the basal value after starting from a high value of blood glucose level, i.e., 200 mg/dL shown in Figure 11. The SMC-based control law depicts far better performance in terms of settling time by acquiring the required glucose level in about 15 s, thus proving the accuracy and robustness of the SMC controller. The reduced value of settling time shows that the desired blood–glucose concentration is achieved earlier and the system becomes stable in less time, facilitating the T1DM patients for have a normalized blood–-glucose level in less time. Simulation results show that SMC also performs well in dealing with the system uncertainties compared to PID and LQR. The robustness of the designed controller against uncertainties can be characterized by introducing a disturbance in the form of a sinusoidal signal.

**Figure 12:**
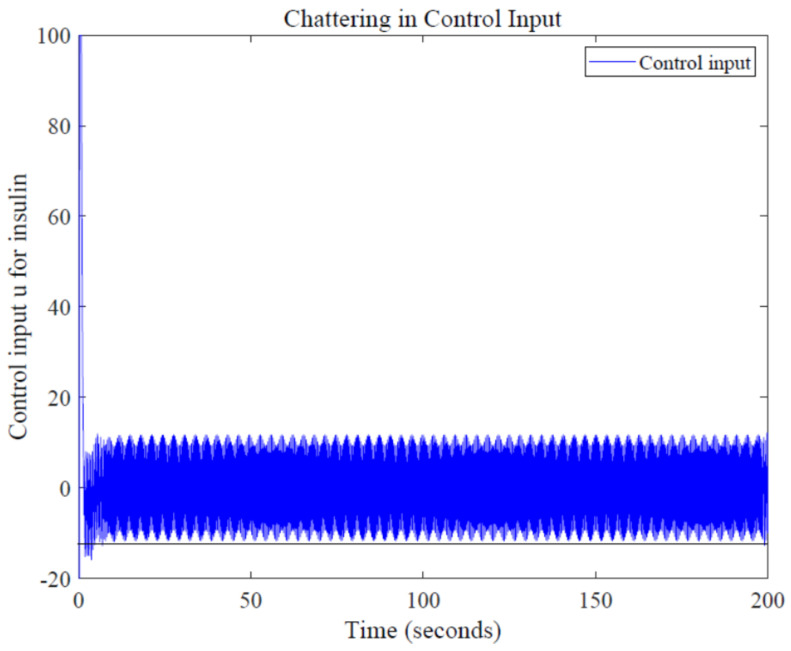
Chattering in control input u.

One major drawback while using first-order SMC is chattering, a common phenomenon which arises due to fast switching of control action around the sliding surfaces. Chattering in control input 'u' can be seen in Figure 12; since high amplitude of chattering is undesirable, low-pass filters can be used along with SMC, which can reduce this high-frequency switching [33]. 

The following section shows a comparative analysis between the two linear control techniques used in this study and SMC with the work presented in [34]. 

## 5. Comparative Analysis

In this section, a detailed and comprehensive analysis is drawn on the basis of time response specifications between the two linear control techniques used in this research work (PID and LQR). In both cases, a blood–glucose concentration of 70 mg/dL is achieved successfully, resulting in the minimum steady-state error. The large transient part of the blood–glucose profile with the PID controller shows that the steady state is achieved in more time, resulting in a large time constant and, therefore, larger settling time. The small transient part of the blood–glucose profile with LQR shows that the steady state is achieved earlier or in less time, resulting in a small time constant and, therefore, small settling time. A small value of settling time shows that the system achieved stability in less time with LQR as compared to PID. The settling time in the case of PID is 430 s and with LQR, it is 113 s approximately, showing better performance in terms of transient and steady-state analysis.

**Figure 13:**
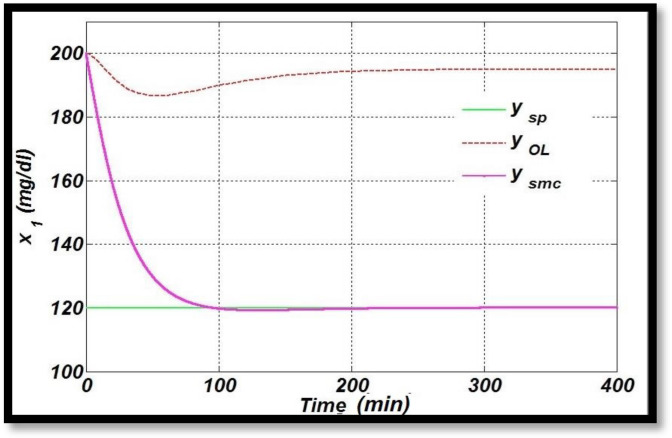
Glucose profile using SMC based on nonlinear transformation.

A comparison of the nonlinear control method, Sliding Mode Control (SMC) used in this research, is drawn with [34], in which an SMC controller is designed based on nonlinear transformation to normalize the high blood glucose level for diabetic patients. In this study, the reference value for blood–glucose concentration is taken as 120 mg/dL, which is achieved at around 100 min with the designed SMC (nonlinear transformation) controller showing a very large settling time in terms of time response specifications, as shown in Figure 13 [34]. Simulation results show that the required blood–glucose concentration is still achieved successfully. The reference value for the optimal glucose level, blood glucose for open-loop configuration and the glucose profile using sliding mode control (nonlinear transformation) in closed-loop fashion are plotted. However, the derived control law using SMC in this research work tracks the desired level of glucose for diabetic patients in an acceptable physiological range of (70 mg/dL to 110 mg/dL) in approximately 15 s, demonstrating the superior performance compared to the linear control techniques discussed above and SMC, based on nonlinear transformation as designed in [34]. The bolus dose is the same, i.e., 200 mg/dL in both the cases. 

## 6. Conclusions

This paper presents control techniques to regulate the glucose levels in patients suffering from diabetes to avoid risks of hyperglycemia and hypoglycemia. The model independent strategy has been considered where the mathematical model describing glucose insulin system was based on experimental data. Discrete time transfer function has been obtained and transformed into continuous domain by the zero-order hold method. Both linear (PID and LQR) and nonlinear schemes (SMC) have been investigated in detail and have been applied on glucose insulin system models. The designed nonlinear control law demonstrated superior performance compared to the linear control techniques in terms of tracking of the desired glucose level (70 mg/dL). Also, the designed first-order SMC was found to yield better results for the estimated model with low sensitivity to plant disturbances and uncertainties. The achieved results are compared with another variant of SMC controller which is based on nonlinear transformation. The derived SMC controller shows better performance in terms of time response parameters. However, chattering in the control input resulted due to the switching action, which is quite evident from the presented simulation results. By applying higher-order SMC techniques along with low-pass filters, chattering can be reduced. 

### Future Action

In future, artificial intelligence-based controllers can be designed to achieve the desired blood–glucose concentration using a reinforcement learning (RL) algorithm.

## Figures and Tables

**Table 1:** Effects of proportional, integral and derivative gains of the PID controller.

Response	Rise Time	Overshoot	Settling Time	Steady-State Error
$k_{p}$	Decrease	Increase	No effect	Decrease
$k_{i}$	Decrease	Increase	Increase	Eliminate
$k_{d}$	No effect	Decrease	Decrease	No effect

**Table 2:** Parameters for the designed PID controller.

$Proportional\;gain\;k_{p}$	−0.000151
$Integral\;gain\;k_{i}$	−0.0000000275
$Derivative\;gain\;k_{d}$	−0.207

**Table 3:** Parameters for the SMC controller design.

Parameters	$c_{1}\;$	$c_{2}\;$	$c_{3}\;$	$k_{1}\;$	$k_{2}\;$
Values	1	2.5	1	6	9

## Data Availability

Not applicable.
